# Diode Laser Assisted Filament Winding of Thermoplastic Matrix Composites

**DOI:** 10.3390/ma3010563

**Published:** 2010-01-20

**Authors:** Fabrizio Quadrini, Erica Anna Squeo, Claudia Prosperi

**Affiliations:** Department of Mechanical Engineering, University of Rome Tor Vergata, Via del Politecnico 1, 00133 Rome, Italy; E-Mails: squeo@ing.uniroma2.it (E.A.S.); claudia.prosperi@gmail.com (C.P.)

**Keywords:** diode laser, laser processing, thermoplastic prepreg, filament winding, composite material, thermoplastic matrix composites

## Abstract

A new consolidation method for the laser-assisted filament winding of thermoplastic prepregs is discussed: for the first time a diode laser is used, as well as long glass fiber reinforced polypropylene prepregs. A consolidation apparatus was built by means of a CNC motion table, a stepper motor and a simple tensioner. Preliminary tests were performed in a hoop winding configuration: only the winding speed was changed, and all the other process parameters (laser power, distance from the laser focus, consolidation force) were kept constant. Small wound rings with an internal diameter of 25 mm were produced and compression tests were carried out to evaluate the composite agglomeration in dependence of the winding speed. At lower winding speeds, a strong interpenetration of adjacent layers was observed.

## 1. Introduction

Thermoplastic matrix composites are substituting traditional long glass fiber composites made of thermosetting matrix for the production of small components in aerospace applications. The main advantages associated with thermoplastic matrix composites are the high shelf life of the prepregs, the higher reliability of the production processes and the simplification of the molding operations. However, typical thermosetting matrix composites processes have to be re-designed for the application to this new class of materials. An important example deals with filament winding process. Regarding thermosetting matrix composites a continuous prepreg is wound on a machine mandrel and subsequently cured in an oven. The typical material adhesivity (known as “tack”) plays an important role in the material consolidation before curing in the oven. Thermoplastic prepregs have no tack properties and have to be joined during the winding operation. The authors have studied a laser-assisted process to allow the thermoplastic prepreg to adhere to the underlying ply simultaneously with its deposition. Use of a laser source to consolidate thermoplastic prepregs is not a novel technology. In 1992, Agarwal *et al.* already proposed the thermal characterization of the laser-assisted consolidation process [1]. They discussed that this kind of technology was able to produce thermoplastic composite parts of excellent quality and structural integrity, combining the advantages of the traditional thermosetting filament winding technology with the faster processing times of thermoplastic prepregs. The experimental apparatus for the laser-assisted consolidation process consisted of a mandrel mounted on a rotational table, a fixed compaction roller, and an 80 W CO_2_ laser. Two ZnSe lenses were used to focus an 8 mm diameter beam into a 6 × 0.5 mm^2^ planar beam, and no winding tension was applied. Experimental tests were carried out on continuous carbon fiber reinforced PEEK prepregs. In 1997, Rosselli *et al.* used a similar consolidation apparatus to process the same material, deepening the effect of the process parameters on the properties of the consolidated composite [2]. They chose to apply a high compaction force in combination with the pre-heating of the compaction-roller: as a result, consolidated composites showed a typical shear strength about 50% of the autoclave processed composites. In 1999, the same consolidation apparatus was used again to process wider prepreg tapes made of carbon reinforced PPS [3]. After that, no further studies about laser-assisted filament winding of thermoplastic prepregs were carried out, even though new interesting materials and laser sources entered the market. In 2003, Wang and Lou proposed a thermal model for on-line laser curing of thermoset composites in filament winding, but no experimental tests were performed [4]. No mention is given to laser-processing of long glass fiber reinforced polypropylene (PP) prepregs, although this new composite material has gained high interest in the scientific world. In 2000, Torres and Bush studied the sheet extrusion and thermoforming of discrete long glass fiber reinforced PP [5]. During extrusion, the long glass fibers were organized into coherent fiber mats which persisted into the solid state, and were able to withstand the deformation process by thermoforming. In 2004, Zampaloni *et al.* presented the stamp thermoforming process which involved supporting the thermoplastic sheet with a bed of heated viscous fluid that applied a hydrostatic pressure across the part throughout the forming process [6]. Experiments were performed on a random oriented continuous glass-mat fiber reinforced PP by means of a hemispherical punch. Dealing with filament winding, numerical simulations are mainly discussed: a first contribution was given by Schlottermüller *et al.* in their study about the simulation of thermal residual stresses in thermoplastic filament winding [7]. Experimental tests were carried out on carbon fiber reinforced PEEK and glass fiber reinforced PP; a gas torch was used as thermal source for the prepreg consolidation in combination with a consolidation roller. Subsequently (2005), this study was extended to evaluate the effect of the tape tension on residual stress and a tape tensioner was added to the consolidation apparatus [8]. Similar experiences were also made by Toso *et al.* who discussed computational simulations and experimental validations for the winding process of glass fiber reinforced PP tapes [9]; also in this case a hot gas torch was used as thermal source. More recently, Antunes *et al.* proposed finite element modeling of thermoplastic matrix composite gas cylinders [10]. Continuous glass/PP commingled fiber tapes were used to produce wrapped pressure gas vessels for domestic applications.

In this study, the authors discuss for the first time the laser-assisted filament winding of continuous glass fiber reinforced PP tapes. A diode laser source was chosen for the composite consolidation, and the easiest winding configuration (*i.e.,* hoop winding) was chosen for preliminary tests. Diode lasers have some industrial advantages compared with other laser systems, namely lower cost and higher efficiency, which permits lower electrical energy consumption. Thanks to their reduced size, an easier integration in production lines is possible. Diode lasers require lower maintenance, guaranteeing a minimum working life of 12,000 hours. Principal disadvantages are related to the low specific power and high spot dimension. Cutting and high speed deep penetration welding are not possible for common metals, but a series of interesting applications are possible for polymer forming and processing. In fact, polymer materials need time to be processed and require low energy; moreover the large spot size is useful to enlarge the laser-processed zone. Experimental tests were performed on small composite rings produced by hoop winding by changing the winding speed but keeping constant the laser power and the focalization condition. Mechanical tests were performed to evaluate the performances of the final filament wound tube portions.

## 2. Experimental Methods

**Figure 1:**
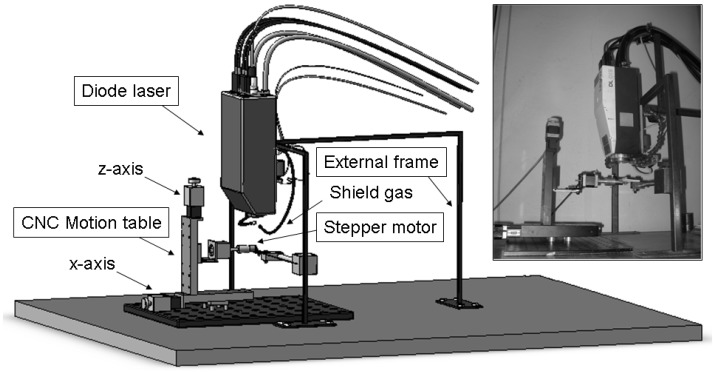
Experimental apparatus for laser-assisted winding of thermoplastic prepregs.

The experimental apparatus for the winding process is shown in Figure 1. A diode laser source was fixed on an external frame as well as a CNC motion table for the sample movement along the x and z direction. A stepper motor was mounted on the motion system so as to provide a rotational speed to a steel tube with the external diameter of 20 mm. The adopted laser was a diode laser (Rofin-Sinar, DL 015) with a 940 nm wavelength and a rectangular spot (3.8 × 1.2 mm^2^) due to the superposition of two different rays, each one coming from a 750 W emitter diode. A 63 mm long focus lens was used to maximize the depth of field. A flux of nitrogen gas was used to protect the lens during working.

**Figure 2:**
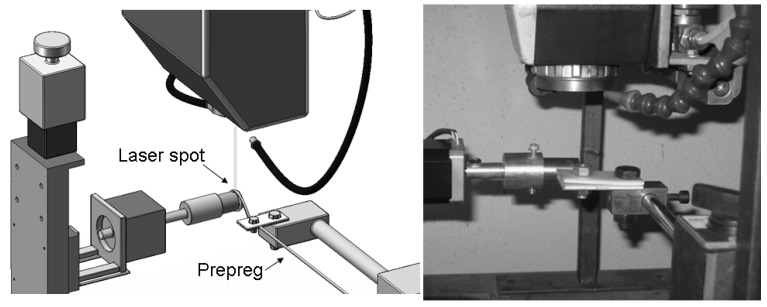
A detail of the consolidation apparatus.

Figure 2 shows a detail of the consolidation apparatus regarding the processing area. A narrow prepreg tape was wound around the steel tube (*i.e.,* the mandrel) and was forced to pass through two polyethylene sheets mechanically clamped together. Due to the friction of the two polyethylene sheets, a consolidation force was applied to the prepreg during winding.

In the experimental procedure, the prepreg tape was wound for a round on the mandrel and shaped by means of a heater. That operation was made by hand, and was necessary for the subsequent positioning under the laser source otherwise the tape stiffness made the laser focalization impossible. After clamping the mandrel to the stepper motor, the non-wound part of the tape was placed between the polyethylene sheets that were clamped in turn. In that condition, the laser-assisted winding process could start: the laser spot was focused on the wound prepreg by means of the z-axis of the CNC table. The laser power was fixed at 50 W and 3 complete revolutions were made without changing the distance from the laser focus. After that, the laser beam was focused again on the external surface of the wound prepreg and other 3 revolutions were performed with the same laser power and rotational speed. Different winding tests were made by only changing the winding speed (14.4, 16.2 and 18 deg/s) because of the very high sensitivity to the laser power: three samples were produced for each winding speed. Higher power values easily led to material degradation or excessive melting, whereas lower power values resulted in poor consolidation.

**Figure 3:**
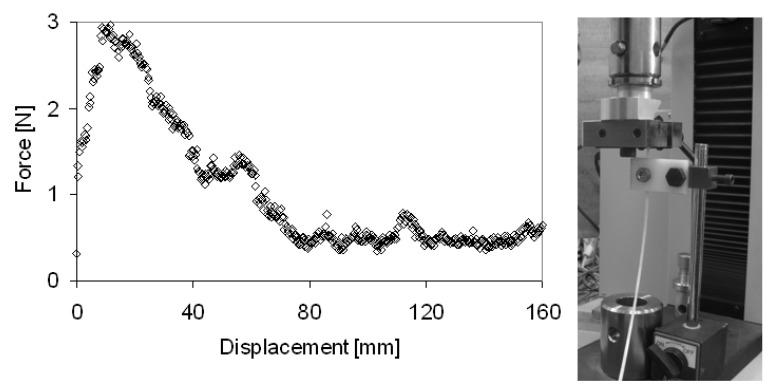
Evaluation of the consolidation force.

In order to evaluate the consolidation force during winding, a simple experimental test was performed. A prepreg tape was clamped to a universal testing machine (Alliance RT/50 by MTS) as well as the tensioner. A constant rate was applied to the prepreg and the friction force was measured. The test rate was fixed equal to the peripheral velocity of the tape during the winding tests, and a small change of the friction force in dependence of the speed rate was observed in the experimented range. For example, Figure 3 shows the friction force at the test rate of 210 mm/min which corresponds to the winding speed of 16.2 deg/s. As expected, a maximum is observed (about 3 N) at low displacements, and a plateau value (about 0.5 N) at higher displacements. On average, a consolidation force about 1 N was observed during the 160 mm long test.

**Figure 4:**
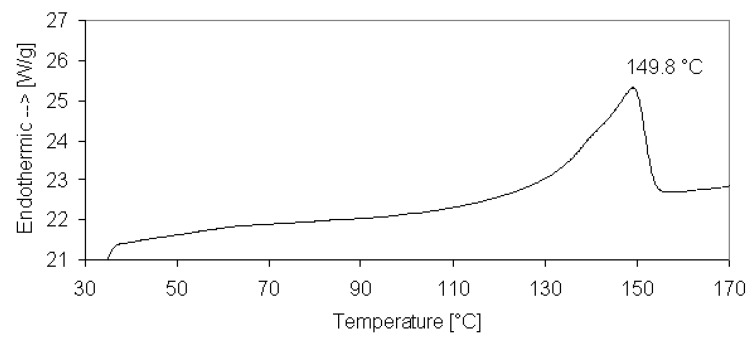
DSC scan of the thermoplastic prepreg.

Unidirectionally reinforced thermoplastic prepregs (by Jonam Composites Ltd, UK) were used to produce the composites. The GPP45 glass/polypropylene prepreg was supplied in the shape of a long tape, 95 mm in width and 0.35 mm in thickness, with the nominal glass content of 45 vol %. Some narrow tapes, 4 mm wide, were cut from the 95 mm tape and used for winding. As the laser spot was focused on the prepreg surface during laser processing, the maximum axis of the spot was aligned with the tape width, so as to cover the entire tape width by the laser spot. This way, a single laser pass was able to consolidate the entire processed tape. The provided material was qualified by differential scanning calorimetry (DSC): DSC scans (by DSC7, Perkin Elmer) were carried out from room temperature to 170 °C at 10 °C/min. Figure 4 shows the melting peak of the PP matrix for the provided prepreg. As the matrix melting is necessary to obtain a complete composite consolidation, a temperature near the peak temperature has to be reached during laser heating. High temperatures would result in an excess of the material flow during forming and also in matrix degradation. On the contrary, low temperatures would be not sufficient for the composite consolidation.

Mechanical performances of the wound composites were evaluated by means of compression tests which were performed orthogonally to the cylinder axis at the test rate of 10 mm/min up to the maximum displacement of 10 mm.

## 3. Experimental Results

**Figure 5:**
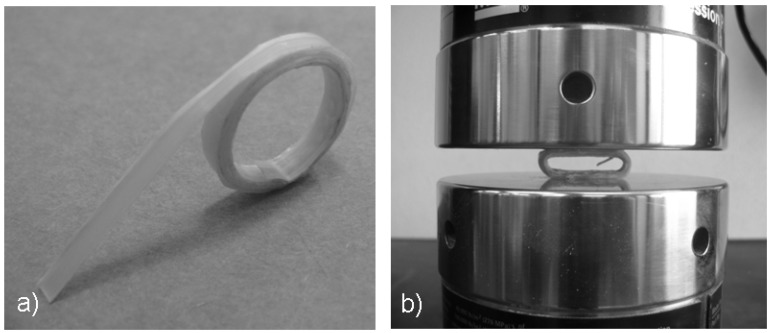
(a) Wound composite at the end of the consolidation process and (b) during compression.

Depending on the winding speed, different results were obtained in terms of composite aesthetics, thickness and mechanical performances. Figure 5a shows the appearance of a typical consolidated composite (at the winding speed of 18 deg/s). All the consolidated composites showed a good adhesion of the prepreg tape along its revolutions. Evidently, the adopted process conditions (*i.e.,* the low laser power, the focalization condition) were sufficient to cause the polypropylene to melt, and the applied force was sufficient to produce the matrix flow necessary for the consolidation. In particular, a very low consolidation force (on average 1 N) was used in combination with a very low laser power (50 W). Low laser power values are generally applied when a good radiation absorption is exhibited by the processed materials. In this case, not only the prepreg but also the underlying steel tube cooperate for the success of the laser-assisted forming operation. The efficiency of the consolidation phase is clearly shown by the mechanical tests. Figure 5b shows a composite sample during compression: A high deformation is applied to the sample without observing any delamination or break.

**Figure 6:**
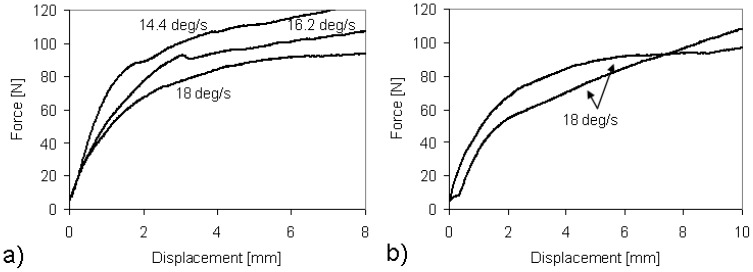
(a) Effect of the winding speed on the compression test and (b) reliability of the results at the highest winding speed.

Quantitative results are shown in Figure 6a and 6b in terms of compression curves. Figure 6a shows the dependence of the mechanical performance of the composite samples on the winding speed. In a previous study, the authors already discussed the results of compression tests on thin composite tubes which were produced by molding the same PP prepregs used in the current study [11]. Even if longer tubes were formed, a similar mechanical behavior was observed. By increasing the winding speed, the compression curve decreases. In all the cases, sample breakage was never observed because of the ductile behavior of the thermoplastic matrix composite. However, more reliable results were observed at the highest value of the winding speed: Figure 6b shows the comparison between two composite samples processed at 18 deg/s. Even if the overall reliability has to be improved, at lower winding speed much lower reliabilities were observed. The material degradation was always avoided, but at lower winding speed the input energy increased and the excess in the material flow made the control of the consolidation process impossible. At high energy inputs, the polymer viscosity is so low that the friction force of the tensioner generates a strong interpenetration of the adjacent layers. Probably the best way to consolidate the prepreg tape is to form the material in a semisolid state: the PP matrix has to be soft enough to make the layer joining possible, but not so much to squeeze away from the consolidated layers.

**Figure 7:**
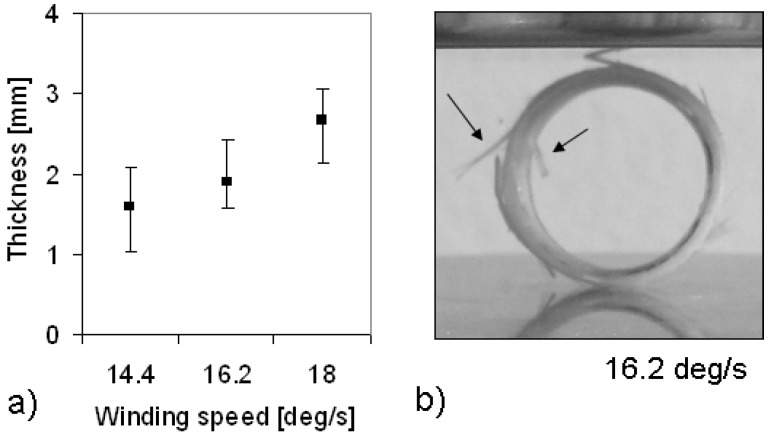
(a) Effect of the winding speed on the composite thickness and (b) appearance of a sample wound at 16.2 deg/s.

This statement is confirmed by Figure 7a and 7b, where the final thickness of the composite samples and the appearance of a composite sample processed at 16.2 deg/s are shown, respectively. Unfortunately, a high thickness variation was observed in the composite rings: a similar variation was also observed along the circumference of each single ring. By increasing the winding speed, the composite thickness increases because of the lower interpenetration between adjacent layers. As the prepreg tape was 0.35 mm thick and 6 revolutions were performed, a maximum composite thickness of 2.1 mm would be expected in the case of a perfect joining between adjacent layers without interpenetration. However, due to the lack of uniformity of the provided tape, the non perfect circular shape of the first tape revolution, and the tape continuity, an average thickness about 2.6 mm was measured in the case of the composite sample processed at 18 deg/s. In this case, the structure of the superimposed layers was still visible through the thickness of the consolidated sample. By decreasing the winding speed, the layer interpenetration increases and the thickness decreases. The structure of the tape is already not visible at the winding speed of 16.2 deg/s, but the excess of the matrix flow is revealed by the emergence of the glass fibers from the sample rims (Figure 7b). Even if the fiber protrusion does not affect the mechanical performances, that occurrence should be avoided during processing. Probably the best processing strategy could request the modulation of the consolidation force in dependence of the winding speed.

## 4. Conclusions

The laser-assisted consolidation of glass reinforced polypropylene tapes by means of a diode laser is a feasible process. A very low laser power is necessary, in combination with a low consolidation force. By changing the winding speed, a different layer interpenetration is generated into the samples with consequences for the thickness and the mechanical properties of the composites. At low winding speeds, even if a good agglomeration is observed, the process is less reliable due to the very low viscosity of the PP matrix. The application of diode lasers to the winding process of thermoplastic matrix composite could to be optimal due to the combination of several advantages: large spot, low specific power, high radiation absorption. As a result, wide areas can be processed with low time and energy consumption and without generating any material degradation. However, many more tests are necessary to reach a process optimum, and the winding system should be improved. In fact, the obtained reliability is not yet acceptable for any industrial application. In the future development, a particular effort will be made to reduce the large thickness variation, and different cylinders with different fiber orientations will be produced, tested and compared with other manufacturing techniques. This way, the proposed technology could be used to produce typical filament wound structures such as pressure vessels or structural tubes.
